# Supplementary material for: Social information use in adolescents: The impact of adults, peers and household composition
Source: PLoS One. 2019 Nov 21;14(11):e0225498. doi: 10.1371/journal.pone.0225498 (PMC6874082; doi:10.1371/journal.pone.0225498)
Supplement: S1 File — (PDF) [file pone.0225498.s001.pdf]

Supporting Information

for

Lucas Molleman, Patricia Kanngiesser and Wouter van den Bos (2019) *Social information use in adolescents: the impact of adults, peers and household composition*

Supporting Figures

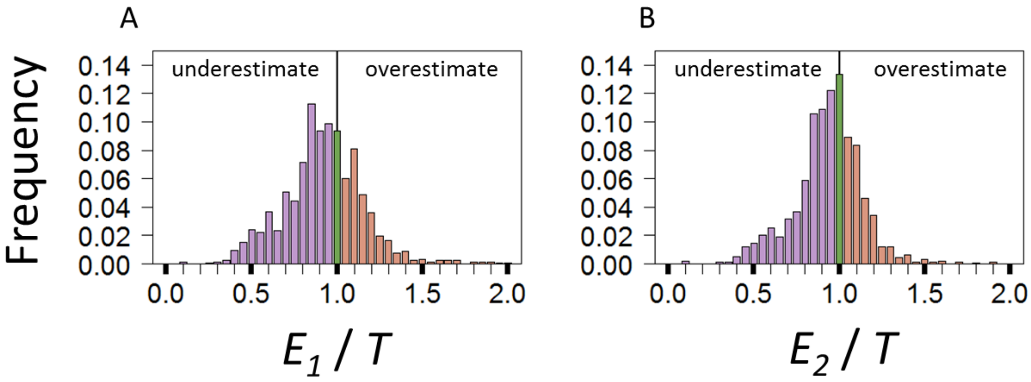

Fig A. Accuracy in first and second estimates. Panels show frequency distributions of participants' estimates before (E_1; panel A) and after (E_2; panel B) receiving social information. For standardization, values were divided over the true value for each of the trials (which varied between 50 and 60 animals). The mean first estimate is 92% of true value (T). This underestimation is virtually identical in magnitude with the % underestimation in a sample of MTurkers from the USA [1], Colombian fishermen and farmers, Dutch university students, as well as British and German teenagers (manuscripts in preparation).

Supporting Tables

	Household type		Test (if applicable)
	**Nuclear**	**Extended**	
Sample size	148	108	
**Demographics**			
Fraction female	0.51	0.48	$\chi 2 = 0.116$, d.f. = 1, P=0.734
Mean age (s.d.)	13.40 (0.89)	13.34	t = 0.530, d.f. = 233.27,
Number of children in household	1.81 (0.48)	1.69 (0.48)	t = 1.859, d.f. = 220.45,
**Religion** (fractions)			
Hinduism	0.93	0.93	
Jainism	0.02	0.03	
Other (Sikhism, Buddhism, none)	0.05	0.04	

Table A. Sample demographics. Our samples from nuclear and extended households did not differ in terms of gender composition, participant age or number of children in their household. Samples were also similar in terms of religious affiliation, with the vast majority of participants identifying themselves as Hindus. Demographic data was obtained along with the consent form sent to parents prior to the experimental sessions.

	Father		Mother	
**Highest educational attainment**	**Nuclear**	**Extended**	**Nuclear**	**Extended**
High school	0.093	0.120	0.104	0.070
Graduate	0.466	0.397	0.602	0.507
Postgraduate	0.364	0.368	0.255	0.341
PhD	0.028	0.043	0.018	0.042
Other	0.049	0.072	0.021	0.040
**Occupation type**	**Nuclear**	**Extended**	**Nuclear**	**Extended**
Services	0.337	0.262	0.106	0.113
White-collar (business, office	0.351	0.332	0.030	0.070
Engineering (IT, electrical)	0.155	0.161	0.020	0.014
Free professions (doctor, teacher)	0.059	0.133	0.185	0.204
Homemaker	0.000	0.000	0.636	0.525
Other	0.098	0.112	0.023	0.074

Table B. Educational and professional background of parents. Numbers in each of the cells reflect fractions. We observe strong similarities across comparison groups (adolescents from nuclear versus extended households) in terms of their parents' highest educational attainment and professional background.

		Aunts / uncles	
		**no**	**yes**
Grandparents	**no**	98	10
	**yes**	106	42

Table C. Sample characteristics with respect to household composition. Numbers in each of the cells reflect numbers of participants in our sample, whose households excluded or included grandparents or aunts or uncles. In the analyses reported in the main text we define 'extended households' as including a cohabiting grandparental generation (regardless of aunts or uncles being present). The results of our analyses are robust to including or excluding aunts or uncles in this definition.

Supporting Text

Normality of the paired differences for the paired t-test mentioned in the Results section of the main text was first assessed by visual inspection, confirming that these differences looked normal. Due to the large sample size, a Kolmogorov-Smirnov (KS)-test detects small but marginally significant deviations from normality (D=0.080; P=0.071). That said, a (non-parametric) paired Mann-Whitney test confirms the results of the reported paired t-test (P=0.040).

For the regressions fitted to participants' mean adjustment S (Table 1, column 1) we followed the same procedure and draw the same conclusion, with a very small (yet borderline significant) deviation from normality (KS-test: D=0.060; P=0.044). For the logistic regressions (table 1 columns 2 and 3), the appropriate (logit) link function deals with the binary outcome variables.

Supporting References

1. Molleman L, Kurvers RHJM, van den Bos W. Unleashing the BEAST: a brief measure of human social information use. Evolution and Human Behavior. 2019 [cited 23 Jul 2019]. doi:10.1016/j.evolhumbehav.2019.06.005

Screenshots of Experimental Materials

The experimental task was completed on a tablet. Here we provide screenshots for each of the screens encountered by participants. We add notes below screens where appropriate.

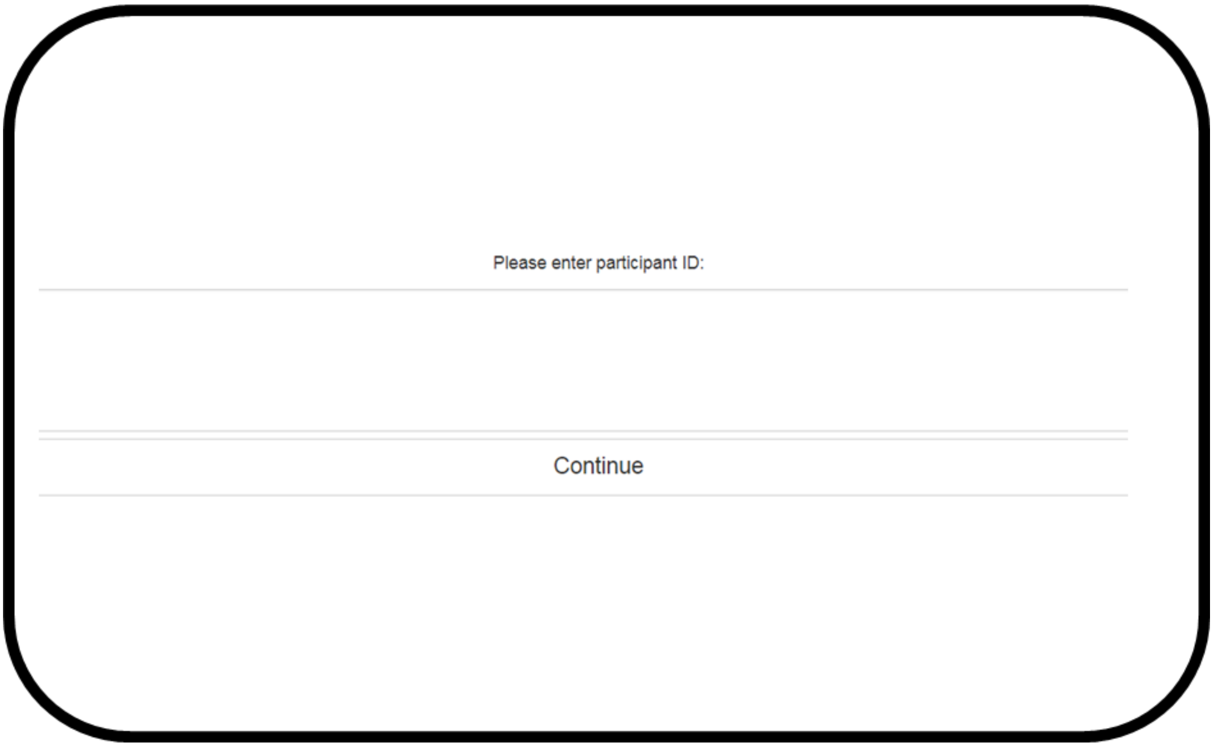

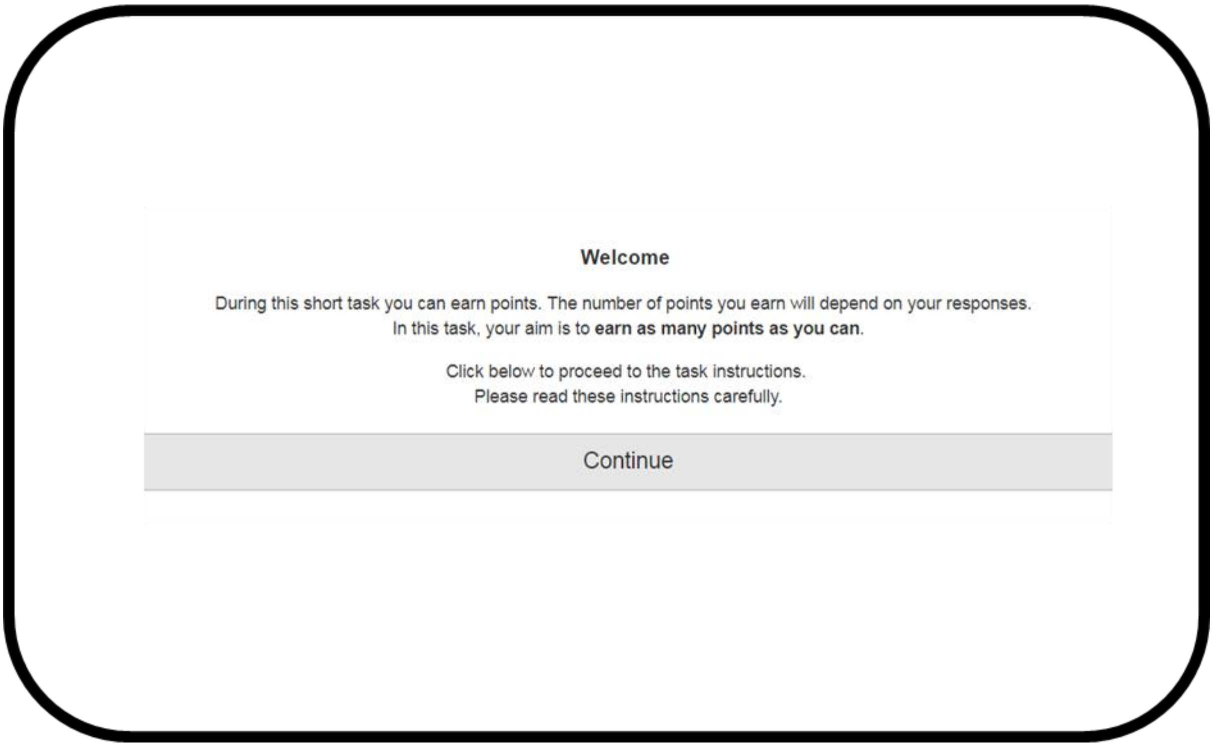

Welcome

During this short task you can earn points. The number of points you earn will depend on your responses.
In this task, your aim is to **earn as many points as you can**.

Click below to proceed to the task instructions.
Please read these instructions carefully.

Continue

Instructions 1 of 5

In this task you have to make a number of **estimates**.
The number of points you earn in this task depends on **how accurate** your estimates are.

Continue

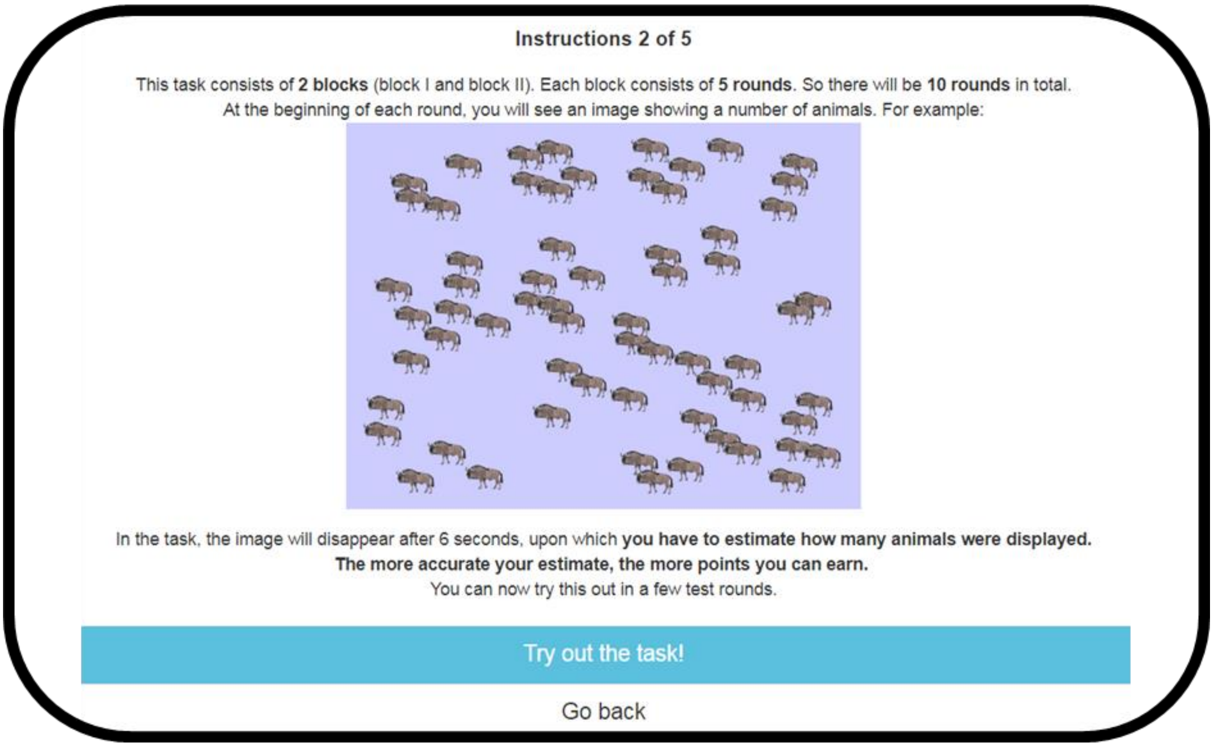

The experimental blocks were counterbalanced across participants.

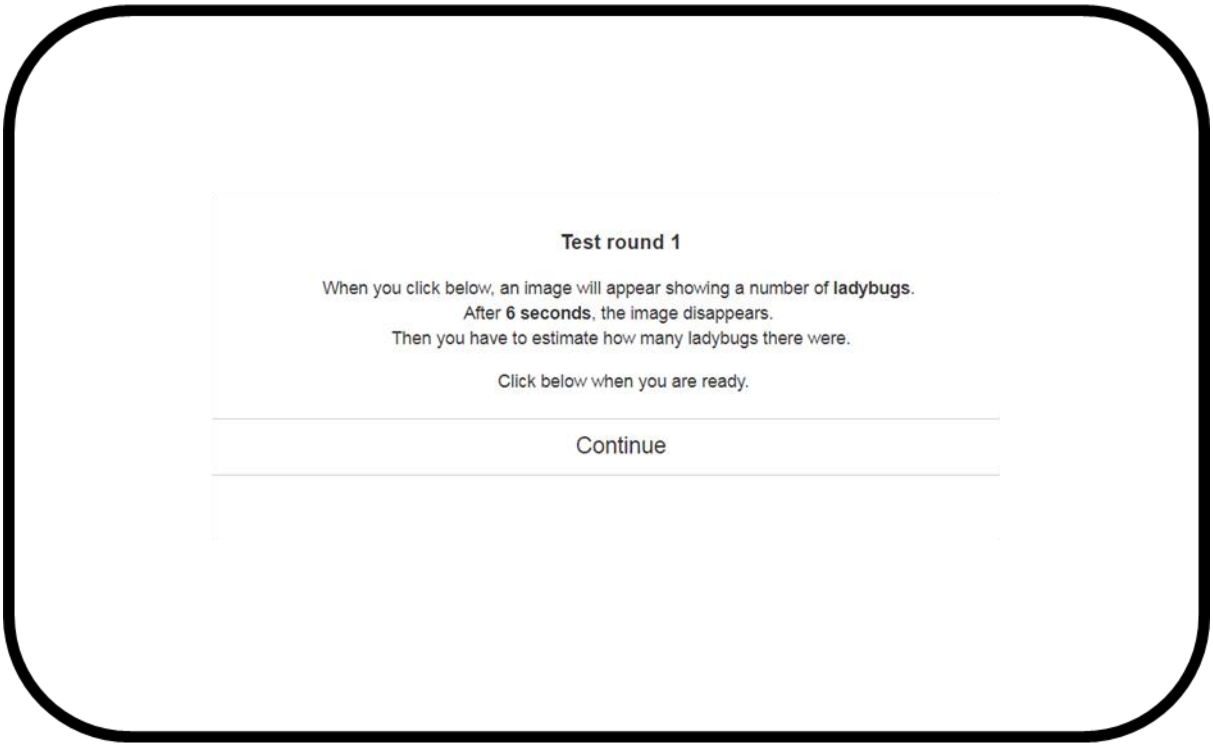

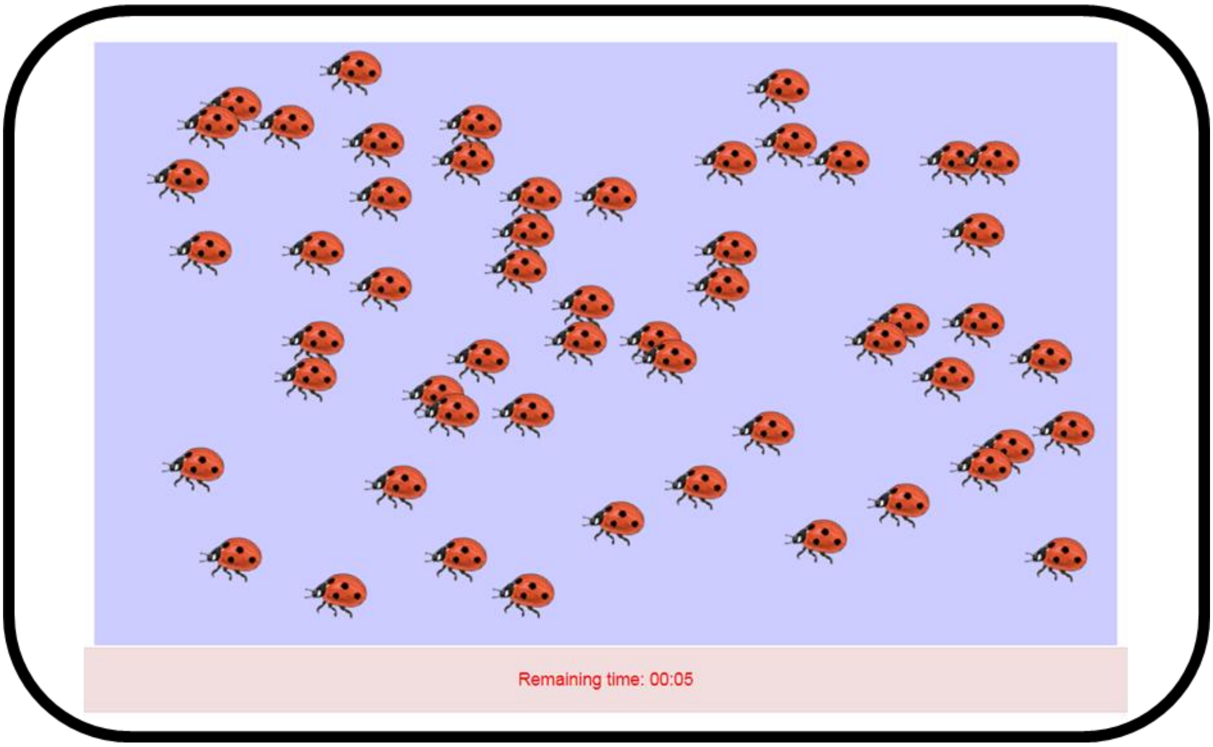

Test round 1

How many ladybugs were shown in the image?

Continue

Test round 1 completed

This is the end of test round 1.
Click below to continue.

Continue

Test round 2

When you click below, an image will appear showing a number of **fish**.
After **6 seconds**, the image disappears.
Then you have to estimate how many fish there were.

Click below when you are ready.

Continue

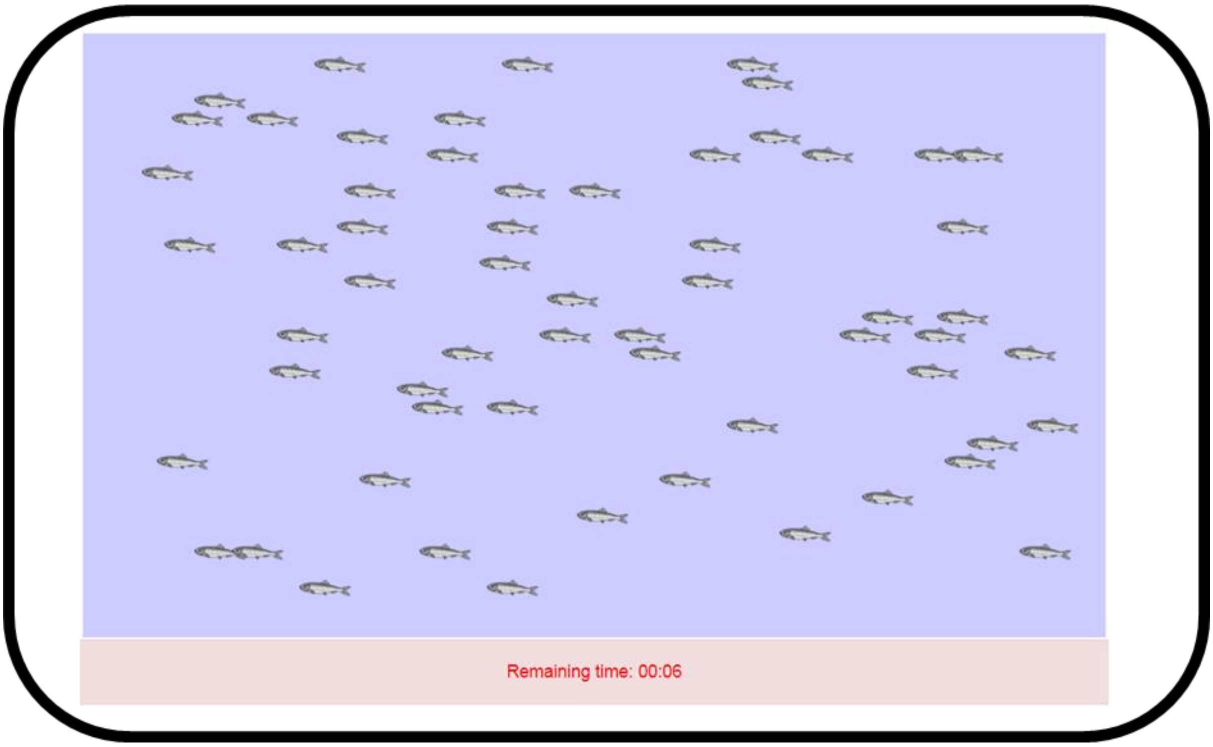
Remaining time: 00:06

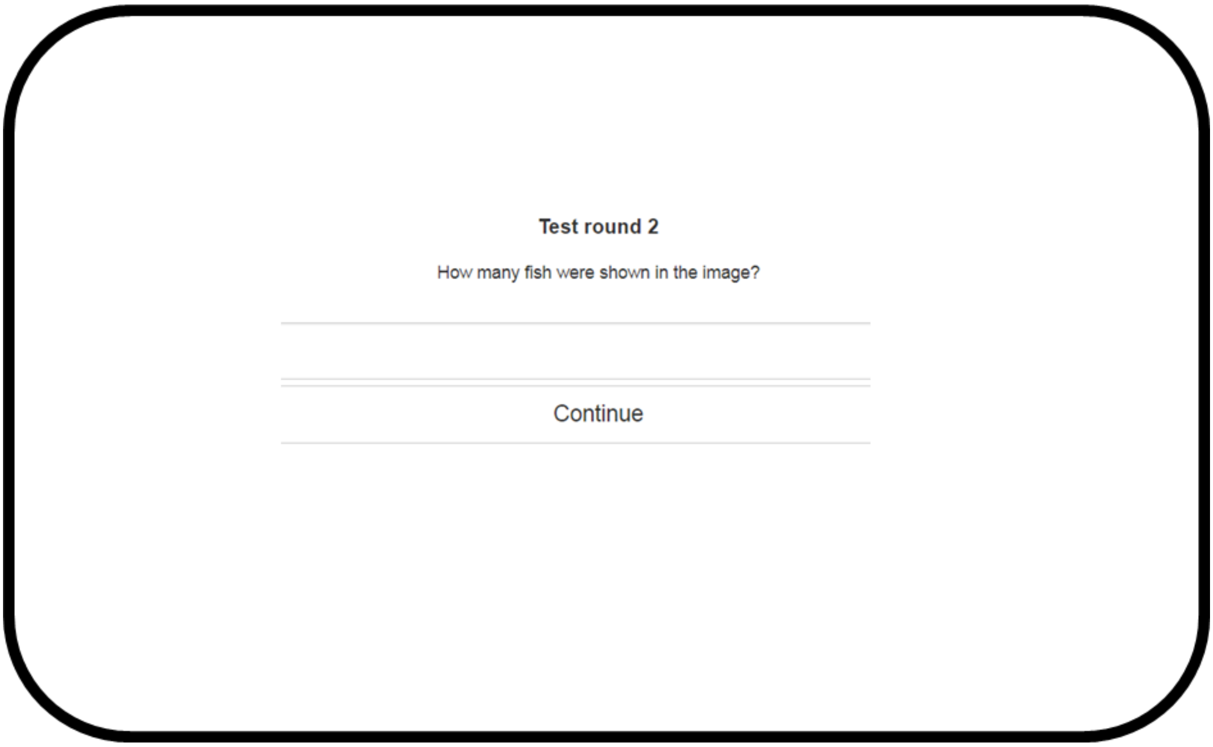
Test round 2
How many fish were shown in the image?
Continue

Test rounds completed

You have finished the test rounds.

Click below to continue to the rest of the instructions.

Continue

Instructions 3 of 5

As you have just seen in the test rounds, the image disappears after 6 seconds.
Once the image has disappeared, you have to enter your estimate of how many animals were displayed.

After you have entered this **first estimate**, you can observe the *first estimate of another participant*.
Over 30 people from Pune recently participated in this study and completed this task.
In each round, you can observe the first estimate of one of these previous participants.

The previous participants saw the same image as you. They also saw it for 6 seconds.
After the image disappeared, they also had to estimate how many animals were displayed.
They could earn more points if their estimate was more accurate.

After you have seen the estimate of the previous participant, you can enter your **second estimate**.
You can enter the same estimate as before, or adjust it as you wish.

After that, the round is over and a new round begins.

Continue

Go back

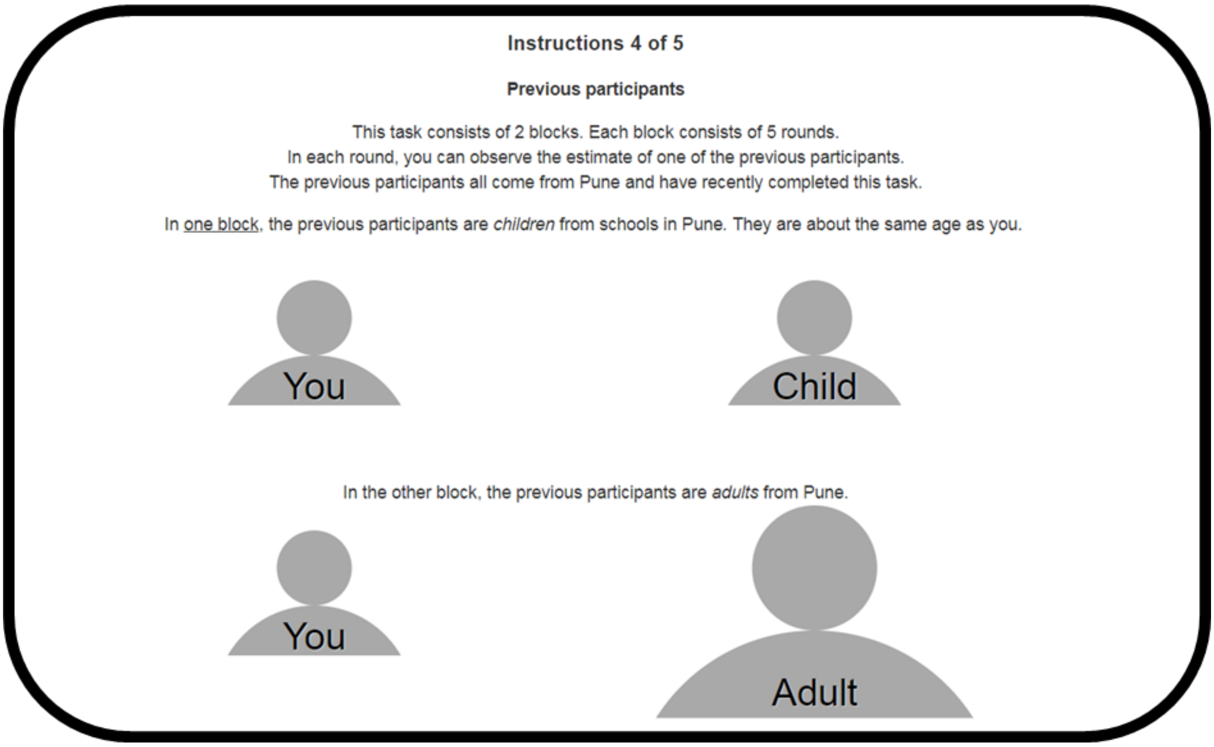

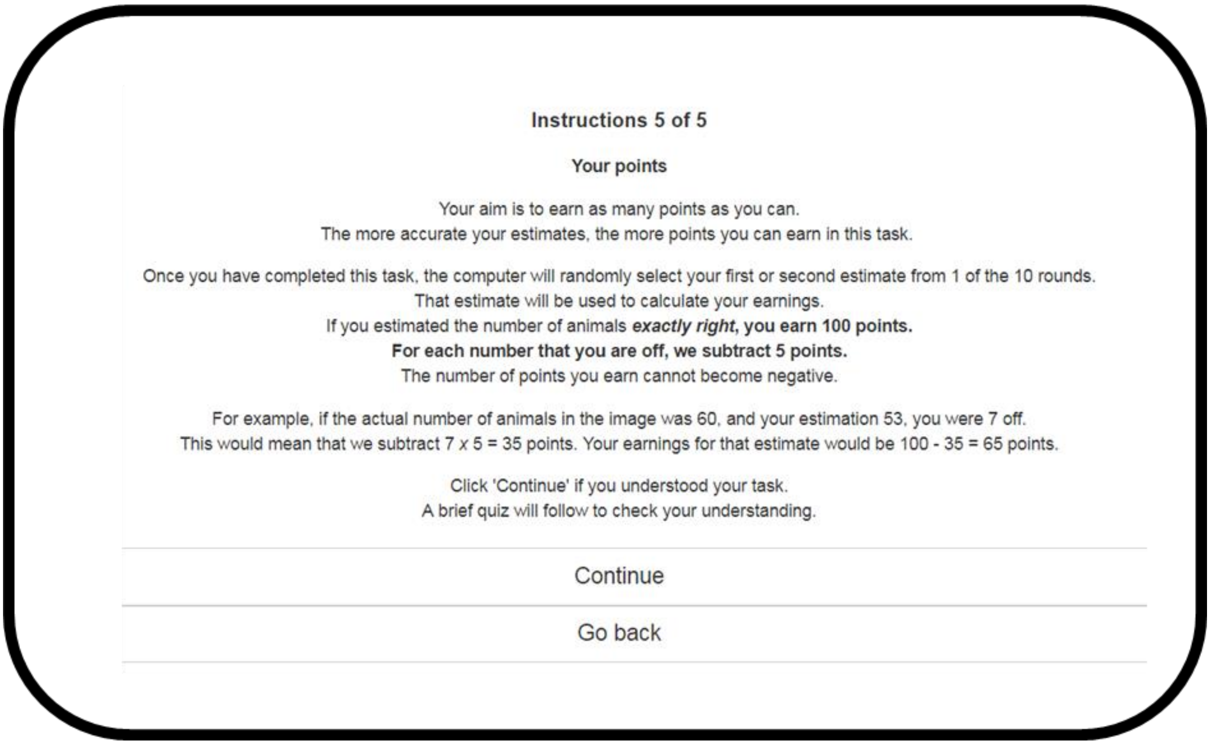

At the end of the experiment, points were converted into stationary items (see Materials and Methods of main text).

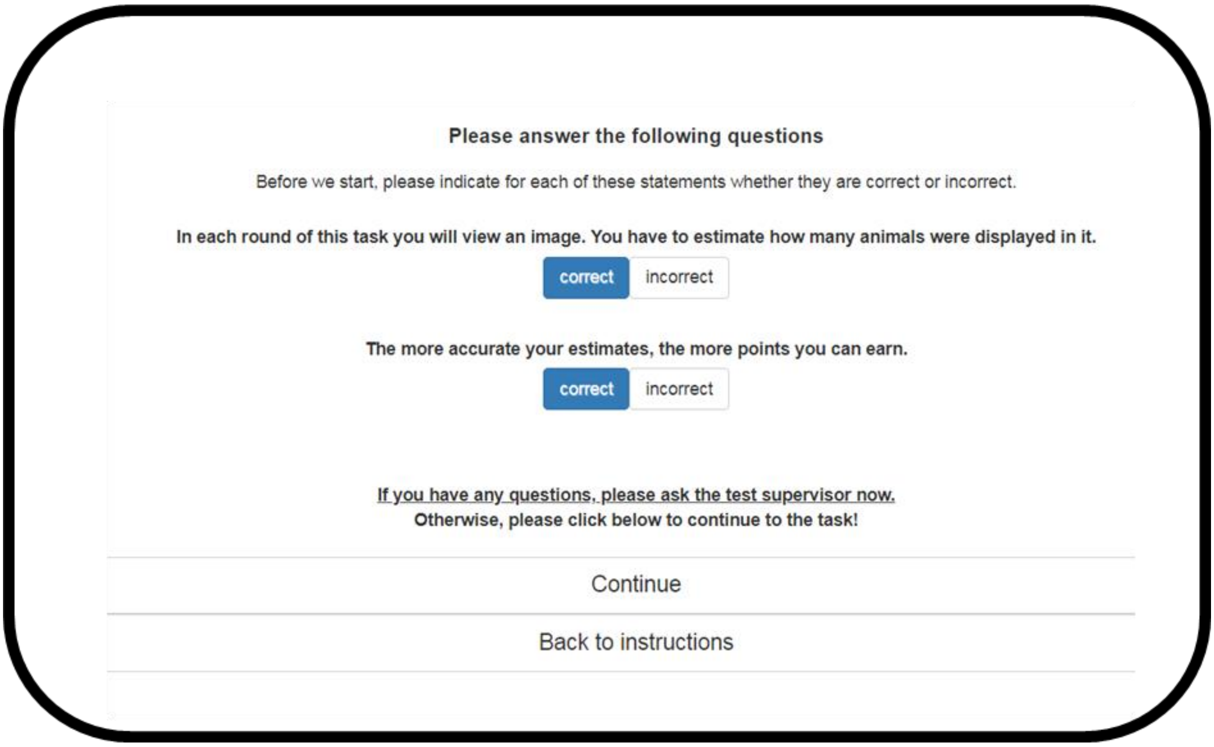

Both control questions needed to be answered correctly before participants could proceed.

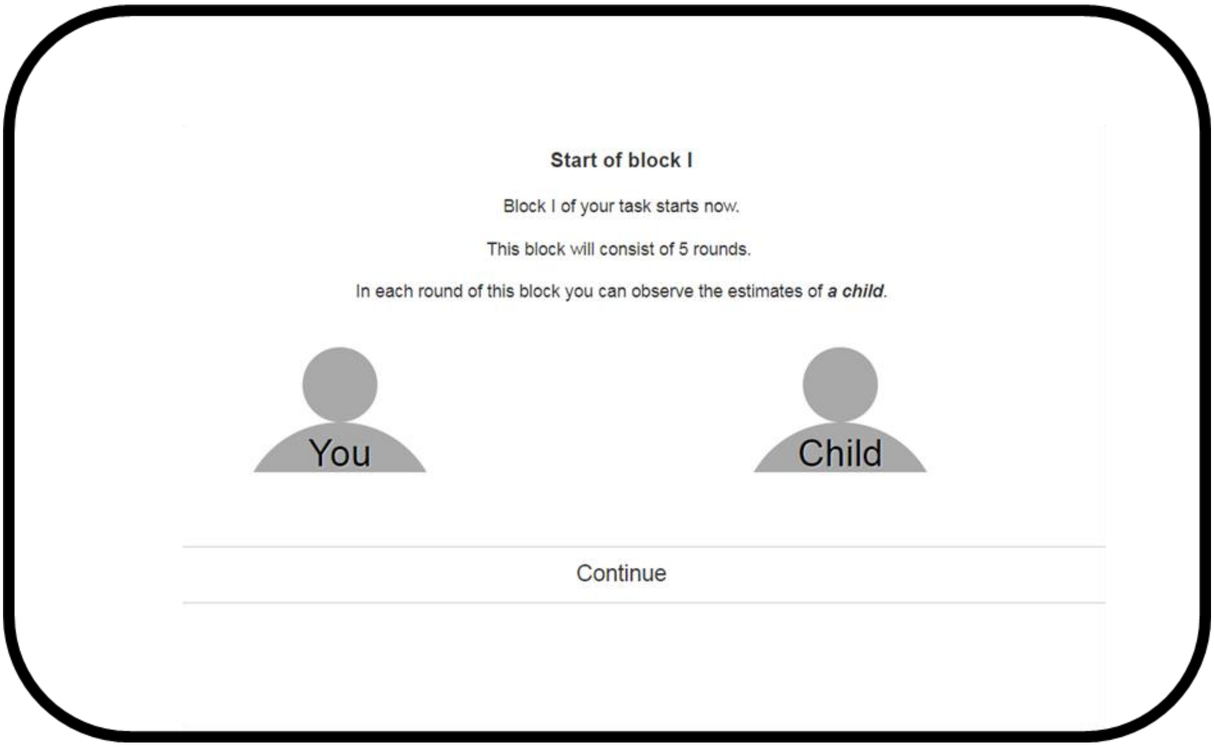

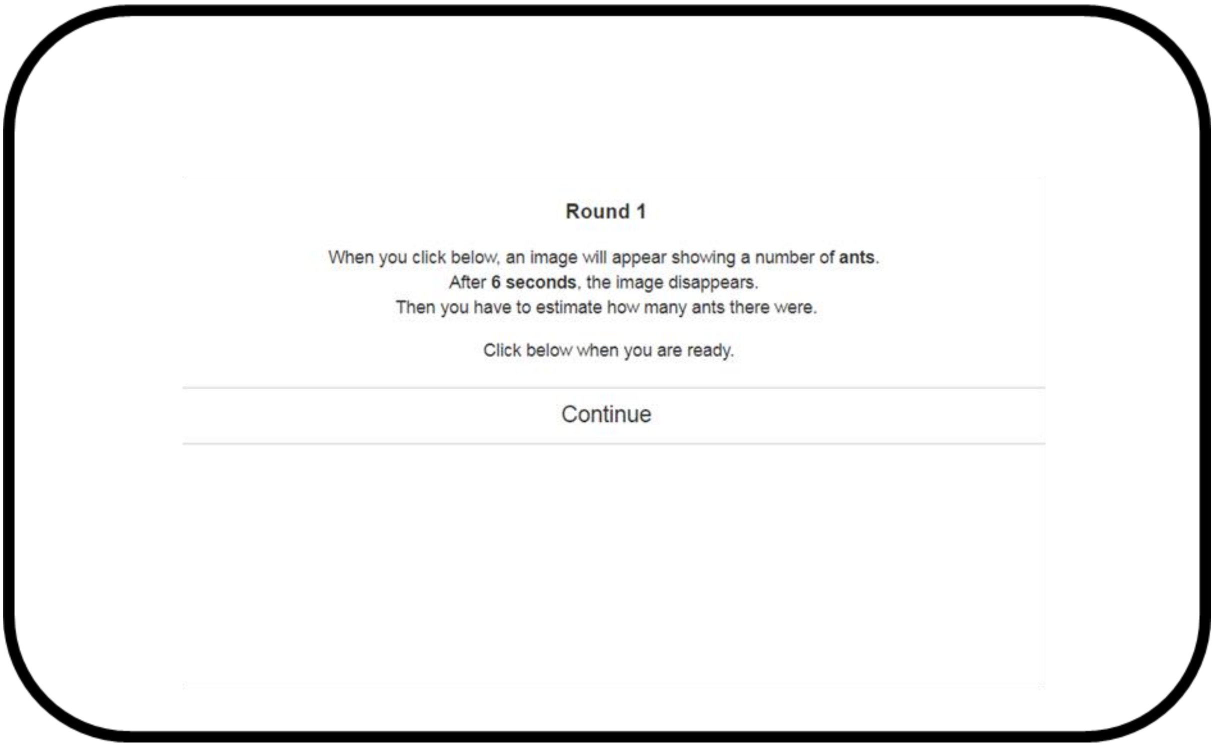
Round 1
When you click below, an image will appear showing a number of ants.
After 6 seconds, the image disappears.
Then you have to estimate how many ants there were.
Click below when you are ready.
Continue

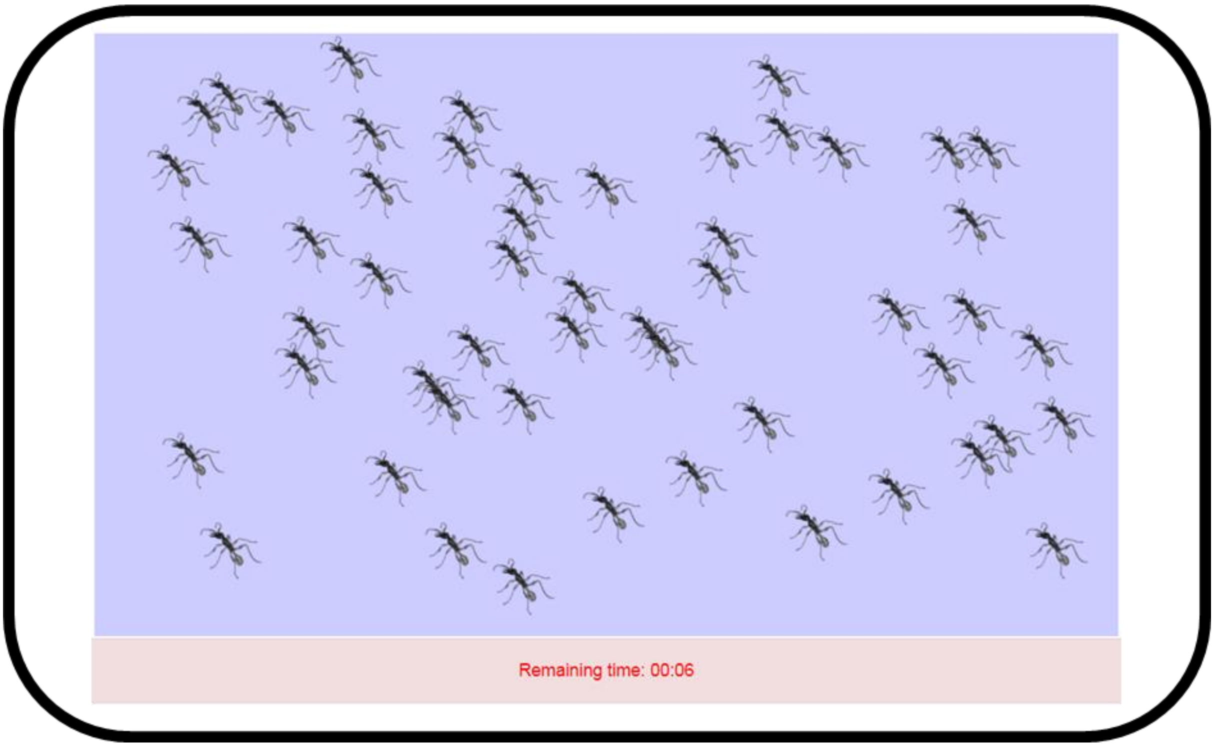
Remaining time: 00:06

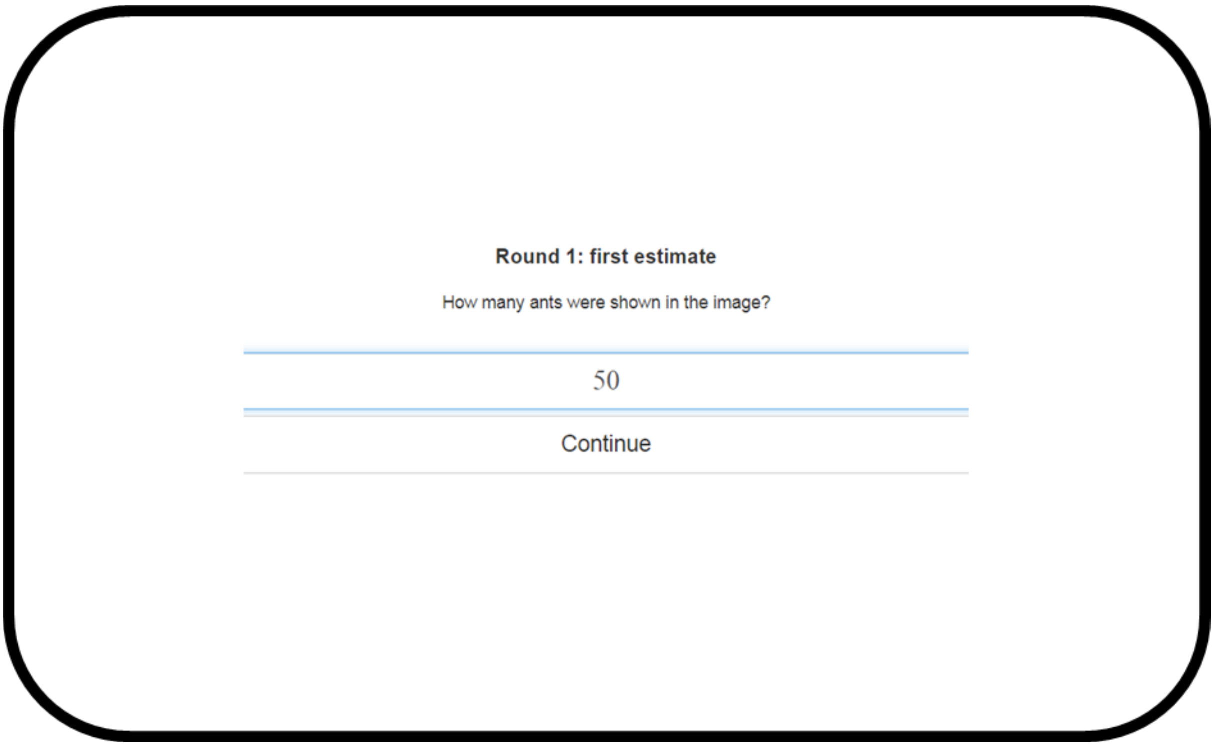

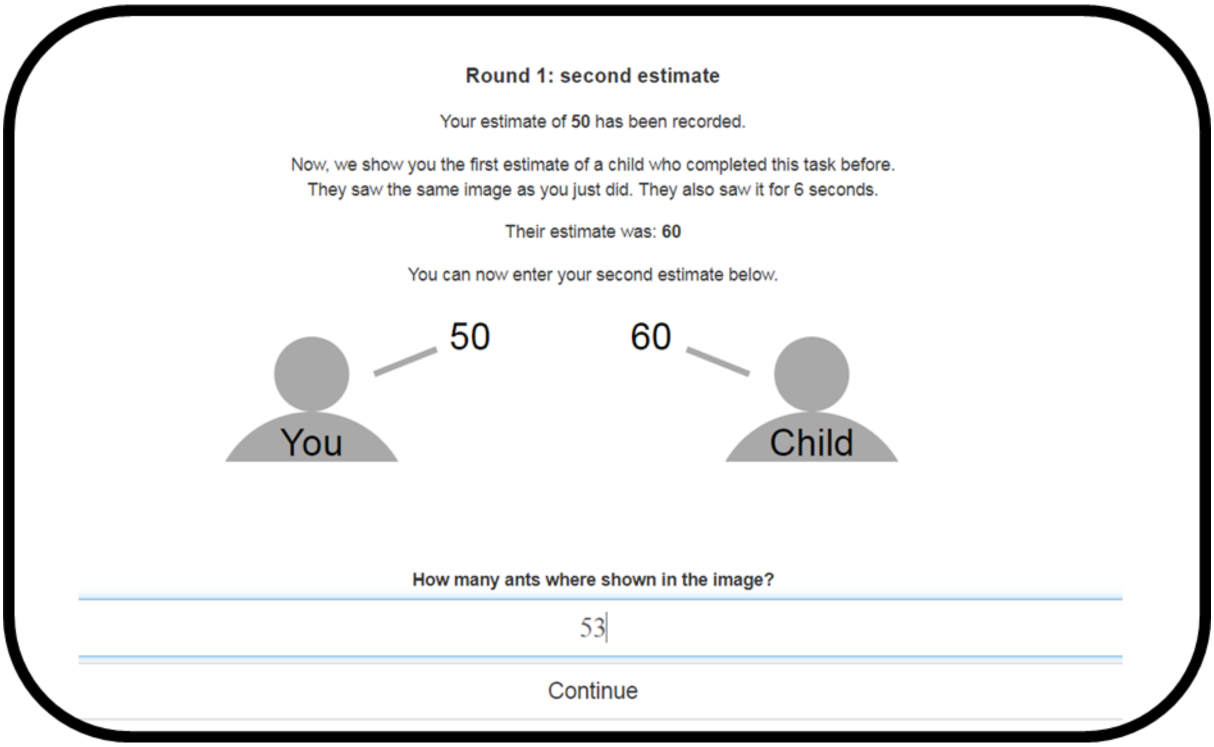

This is the key screen for measurement of social information use. In the round of this example, the participant's first estimate (E_1) was 50, the social information (X) was 60. The participant now makes their second estimate (E_2; in this case 53). For this round, we would calculate $s = (E_2 – E_1) / (X – E_1) = 0.3$ (see Materials and Methods in main text for details).

Round completed

This is the end of round 1.
Click below to continue.

Continue

For rounds 2-5, decision screens for submitting estimates were similar to round 1. Here we just show the stimuli for these rounds.

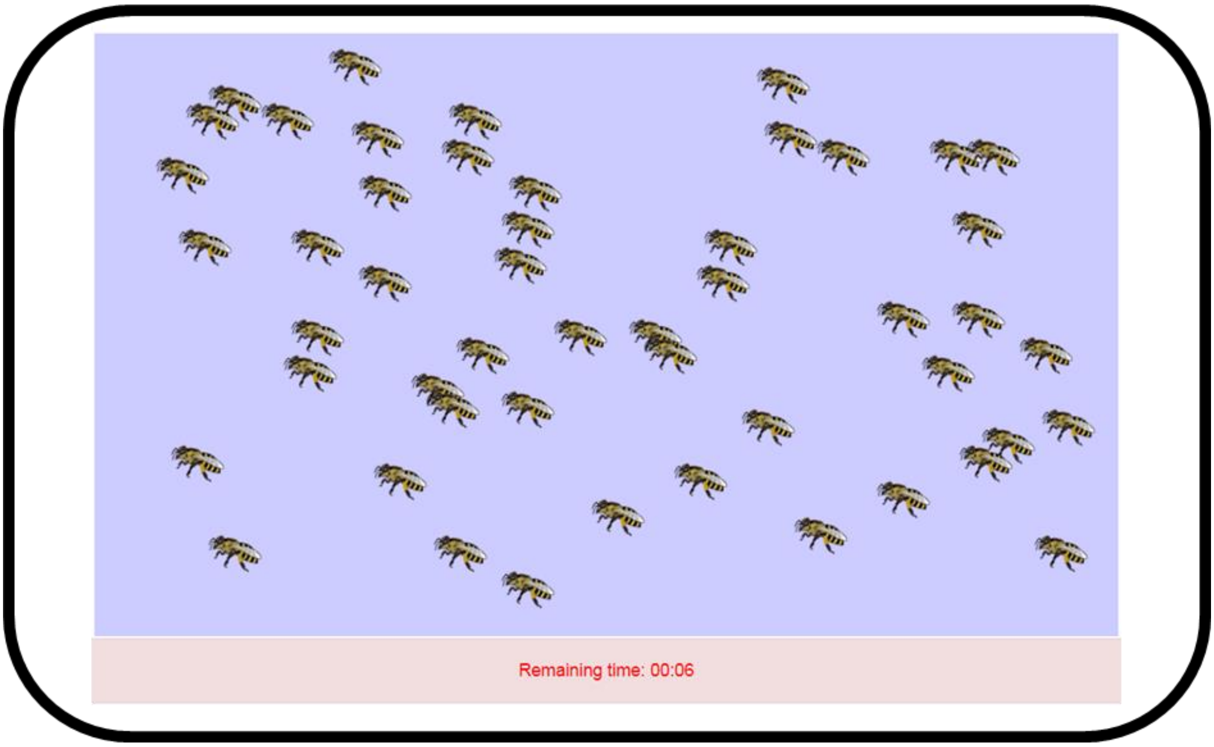

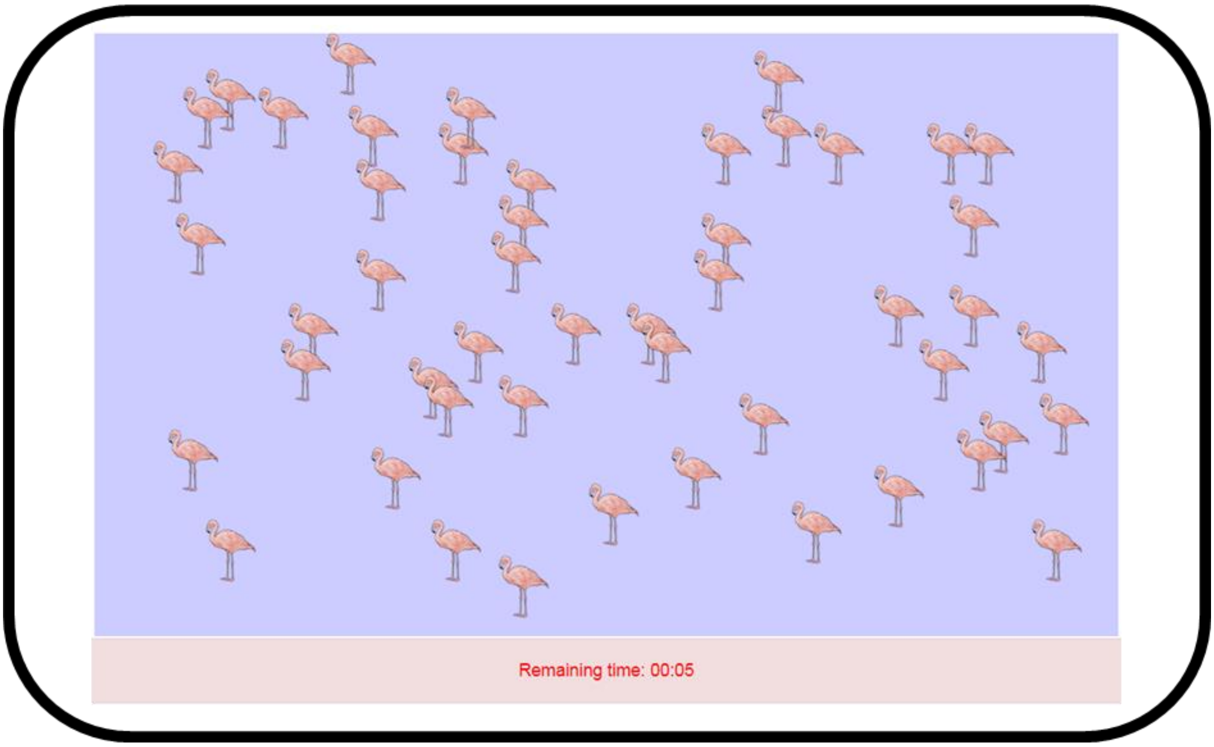

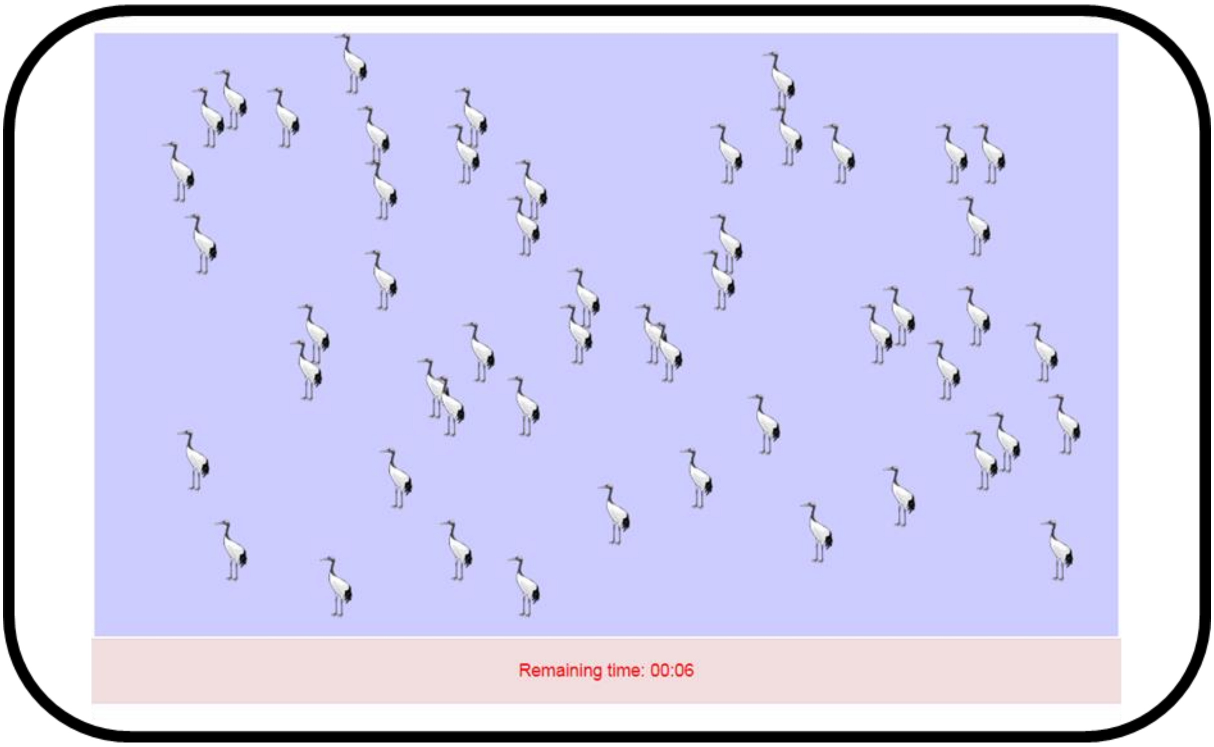
Remaining time: 00:06

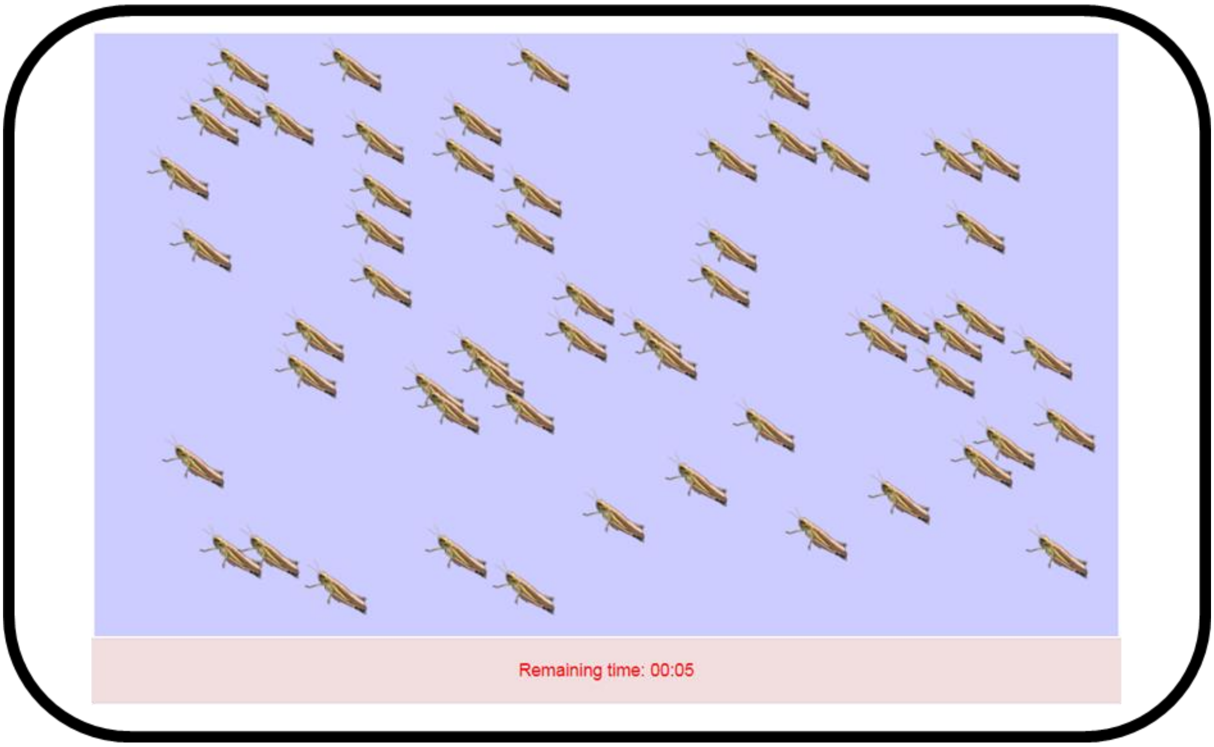
Remaining time: 00:05

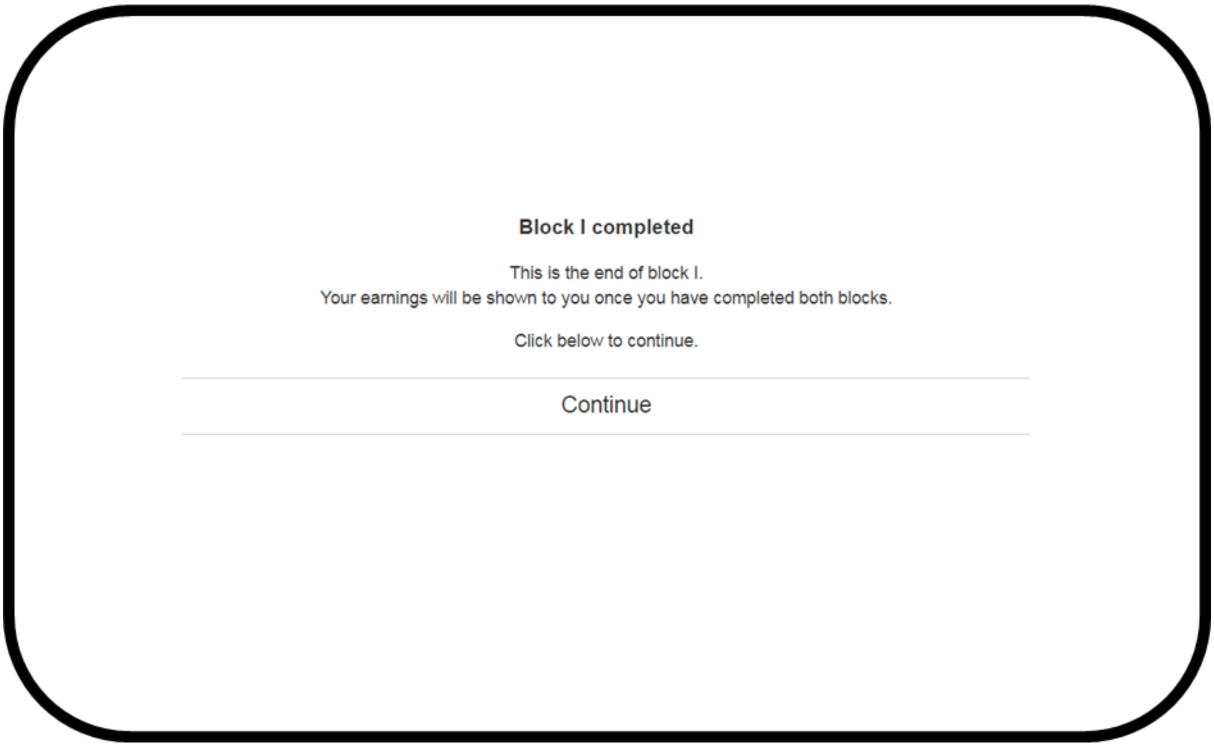

When participants reached this screen, they had completed the first block. The next block then started. As stated above, these blocks were counterbalanced between participants.

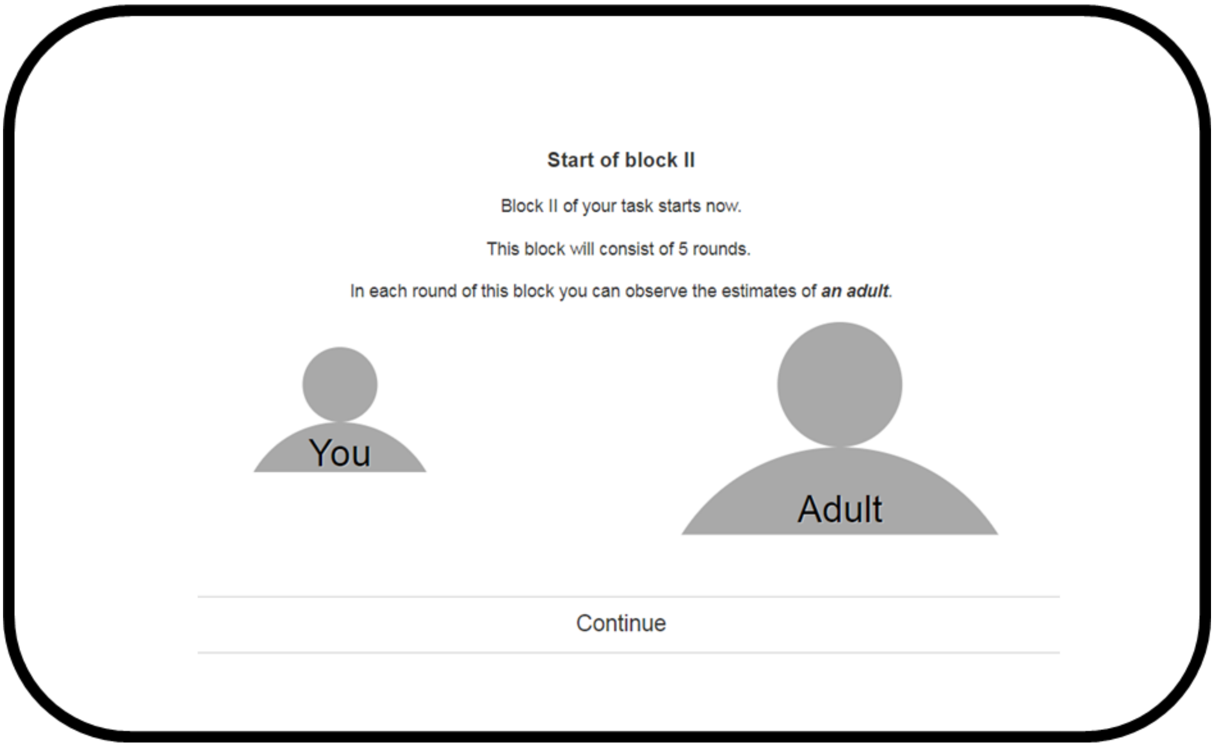

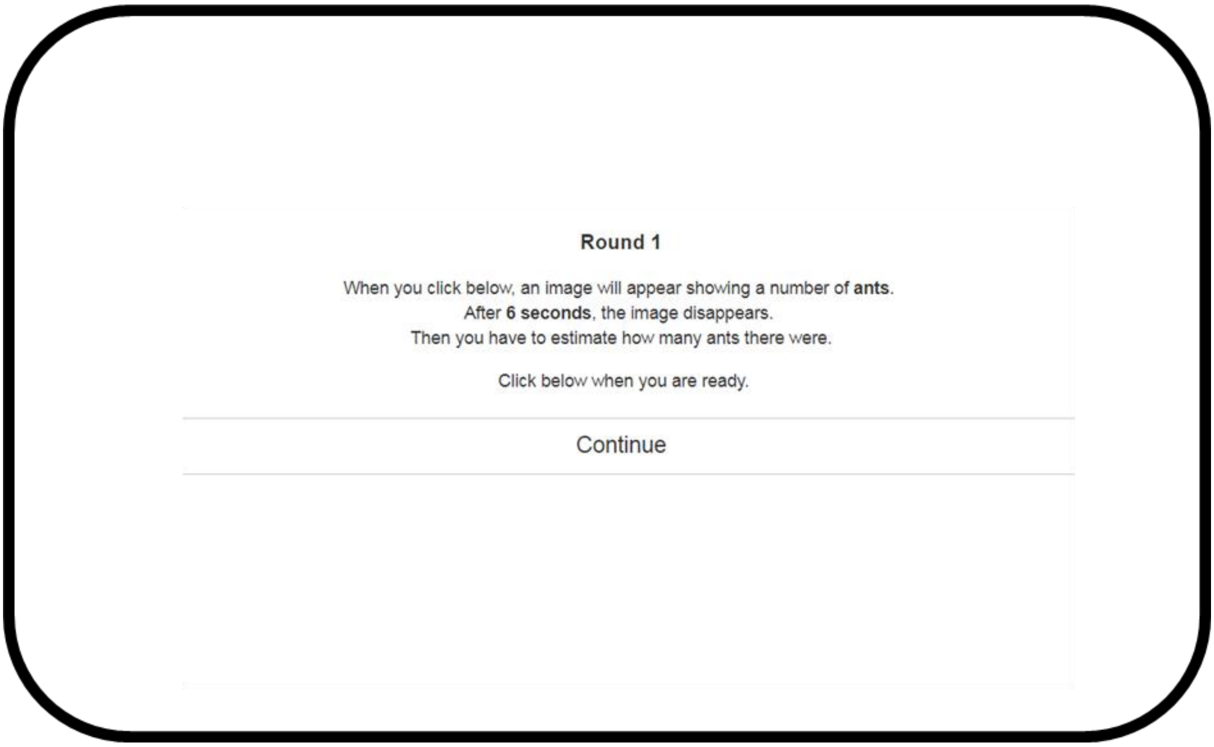

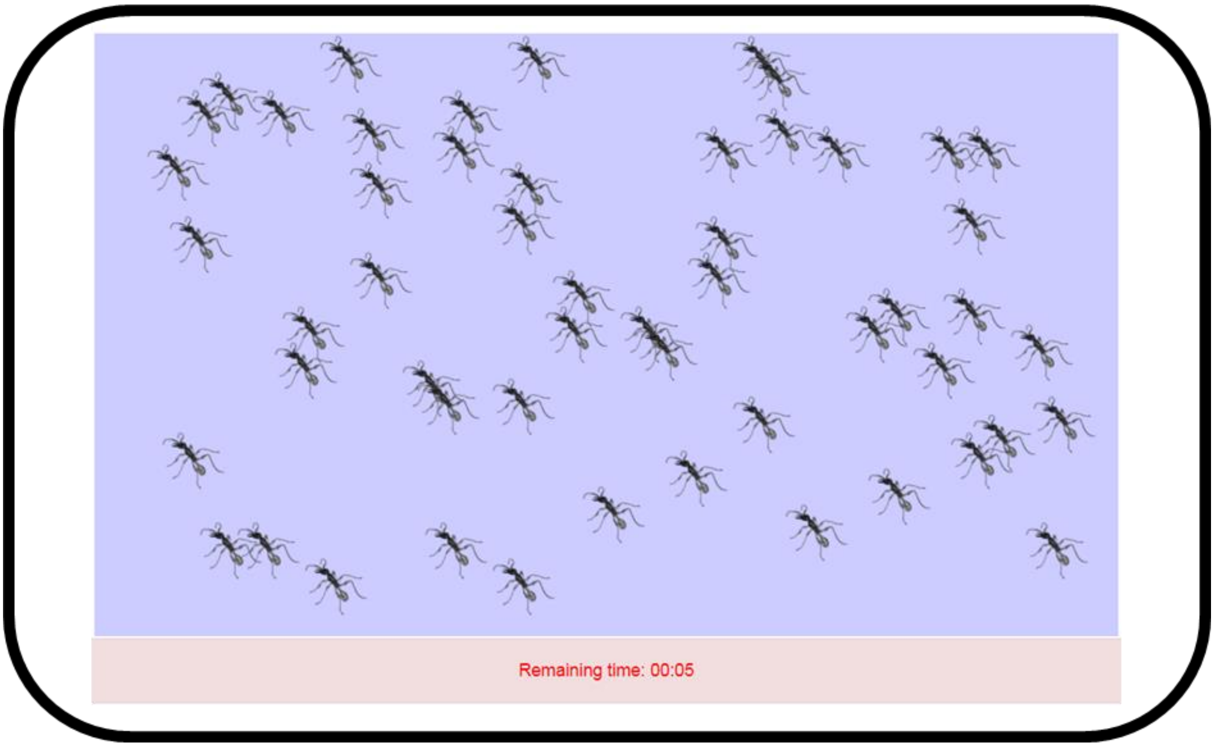

Stimuli for the rounds of the blocks were very similar; see above for screenshots.

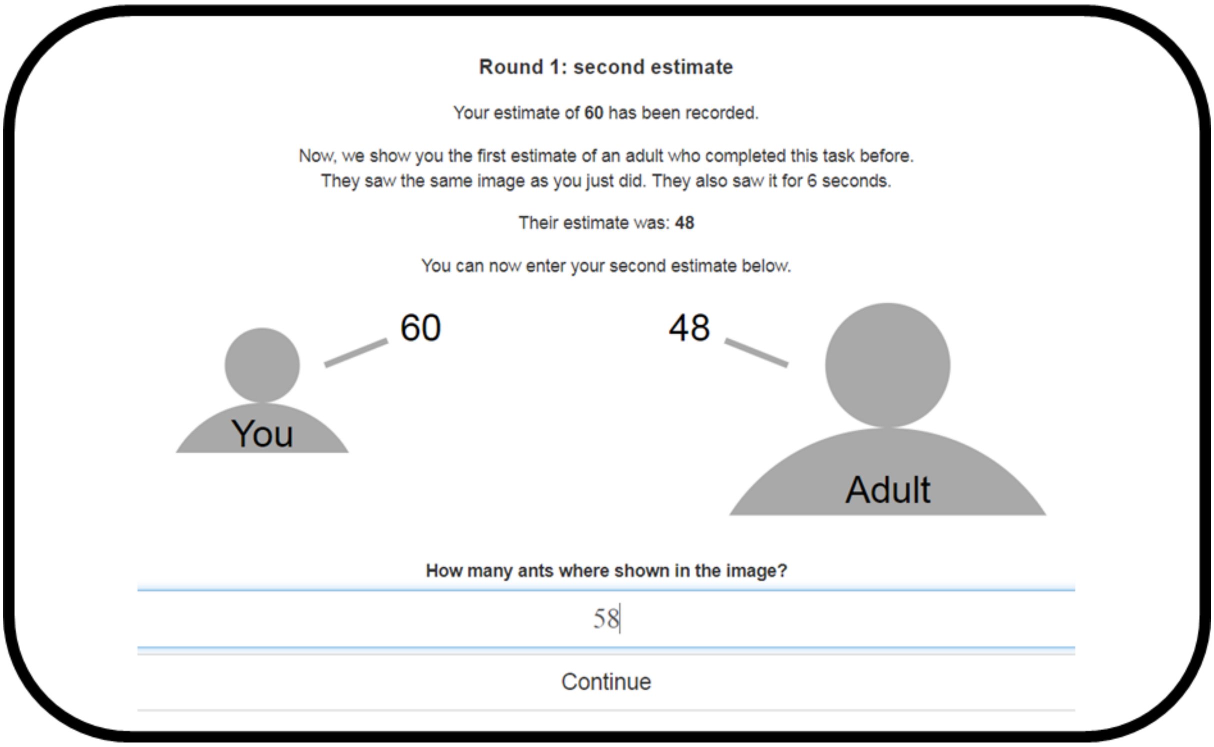

The key screen for measuring social information use. NB: social information was always in the direction of the true value (*T*). In this example, the participant's first estimate was higher than *T*; the provided social information (*X*) was lower (see above for a case where the participant's first estimate was lower than *T* (and the provided social information, *X*, higher)

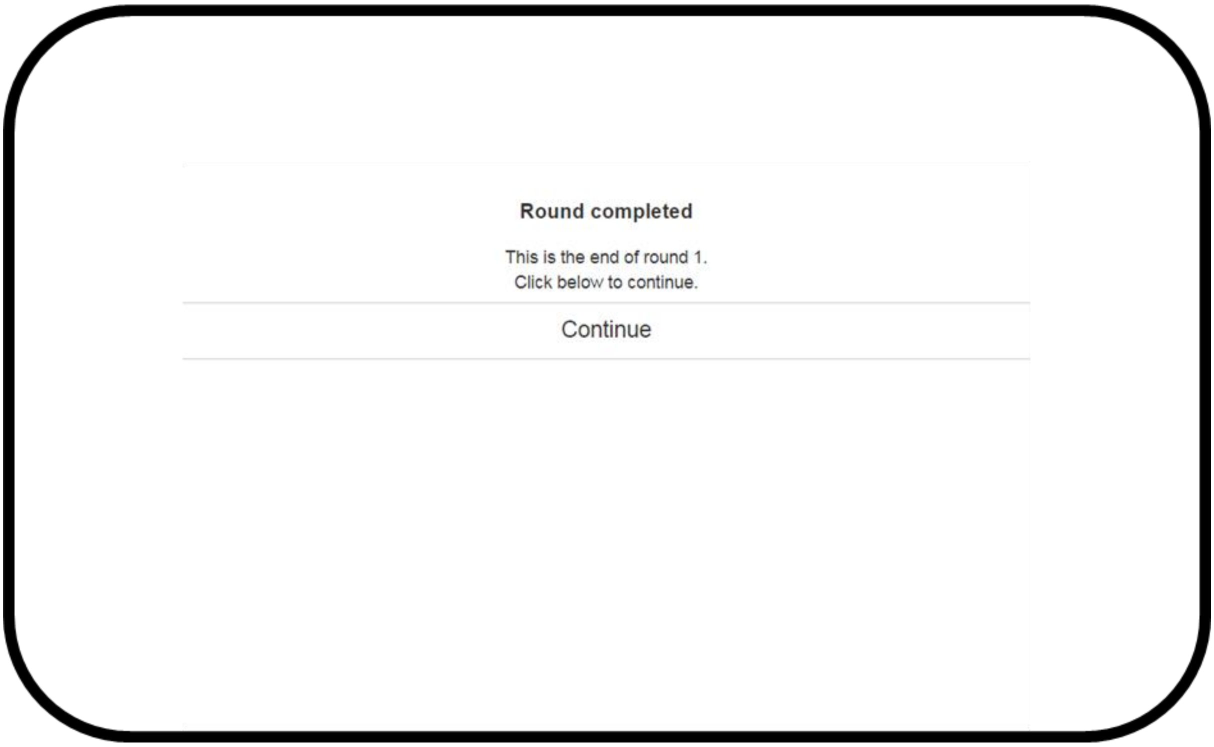

End of task

You have now completed this task. Click continue to see your bonus.

Continue

Earnings

This is the end of this task.

Your points are calculated as follows.

The computer randomly selected **your first estimate from block II, round 4**.
In that round, you estimated how many **cranes** there were in the image.

Your estimate in that case was **65**.
The actual number of cranes in the image was **52**.

This means that your estimate was **13** off the actual number.
As a consequence, we subtract 13 x 5 = 65 Points.

You have earned 100 - 65 = **35 points**.

Thank you for your participation.
