# Supplementary material for: Social information use in adolescents: The impact of adults, peers and household composition
Source: PLoS One. 2019 Nov 21;14(11):e0225498. doi: 10.1371/journal.pone.0225498 (PMC6874082; doi:10.1371/journal.pone.0225498)
Supplement: Attachment — Submitted filename: MKB - Rebuttal.docx [file pone.0225498.s002.docx]

**Response to reviewers**

For: Molleman, Kanngiesser and Van den Bos: “Social information use in adolescents: the impact of adults, peers and household composition” (PONE-D-19-24671)

Dear Dr. Nielsen,

Thank you for your decision letter. We were very happy to hear the positive comments by yourself and the reviewer. All specific review items were answered with ‘Yes’, so in the below Response to Reviewers we focus on addressing the reviewer’s Comments to the Authors. We have printed the reviewer comments in black and our responses in blue. In these responses, cited line numbers refer to the manuscript document *with* tracked changes.

Furthermore, as requested in the Journal Requirements listed on the top of your email, our revision adheres to PLoS One style requirements and file naming. We included a caption for our Supporting Information files, and updated in-text citations to match these. Finally, our ethics statement (Methods section and submission information) now mentions that all data was collected by a local researcher, and that heads of school gave permission to conduct the study in their school and handed out consent forms to parents.

Kind regards,

Lucas Molleman, Patricia Kanngiesser and Wouter van den Bos

**Reviewer #1**: This study examined adolescences’ whether social information use is influenced by the age of the model (peer vs adult) and/or participants’ household demographics (nuclear vs extended family). Using a number estimation task, results indicated that adolescents showed high levels of social information use, particularly when the source was an adult, while household composition had no effect on social information rates. There was also evidence of heuristics whereby adolescents demonstrated high rates of directly copying others estimates.

Overall, I really liked this study. In general, I had very few criticisms of the manuscript – I thought it was well written and I was very pleased to see that the participant sample was from India (and I really appreciated the use of sample-specific references). This is an important step for the cultural evolution literature to expand the range of populations tested beyond Western ones, and not just for comparisons. I was also extremely pleased that the manuscript specifically addressed adolescents. I personally advocate the need for our field to move beyond the young children/adult dichotomy that we seem to be stuck upon. As such, the question and findings are interesting and are clearly presented.

Thank you for these positive comments.

One thing I was interested in was whether the authors validated the task. A recent, growing body of cross-cultural studies have shown that tasks designed for use on Western populations often capture something different when taken to non-western populations. I acknowledge and understand that the BEAST was successfully implemented on a diverse population (from MTURK) – which is excellent and a good indication is translates well. But have the authors shown that the task is capturing social information use in the same way across cultures?

Thank you for bringing this up. By using a minimal social context, the BEAST aims to be applicable across cultures. In recent months we have used the BEAST to measure social information use among farmers and fishermen in rural Colombia, Dutch university students, as well as among (urban) British and German teenagers. The data from these populations replicate many basic aspects of the data reported here: people tend to (*i*) initially underestimate the number of animals, (*ii*) tend to stay with their initial estimate, or compromise towards the social information, and occasionally copy it, (*iii*) often take a weighted average, assigning slightly more weight to their own estimate than to social information.

We agree that in principle, it would be good to expand on these findings more. However, the manuscripts on these experiments are currently still in preparation. In our revised manuscript, we now mention that the magnitude of underestimation (point *i* above), as well as the overall accuracy improvement after observing social information in our data is very similar to the underestimation in these other populations; indicating that manuscripts on these data are in preparation (lines 250-253). Furthermore, in pilot sessions we informally debriefed participants; they indicated that they did not doubt that social information stemmed from actual people, and that they found it potentially useful to improve the accuracy of their estimates (now added in lines 222-225). This makes us confident that social information in our task is used in similar ways across different cultural contexts. Finally, we now cite the papers suggested by the reviewer (see point below), which critically discuss the use of cognitive measures in different populations (lines 340-342).

What language was the task administered in (the screenshots suggest English, but I wasn’t sure if this was the version administered in this study)? If not English, what was the translation process (i.e. back translations)?

The task was administered in English. All participants attended English middle schools and had good proficiency in this language. Instructions were straightforward (printed in full in the SI) and included extensive visual aids as well as compulsory control questions to ensure that participants fully understood their task. Furthermore, a local experimenter was present for all of the data collection and also spoke the local languages (Hindi, Marathi) in case participants had any questions from participants. We mention these points in the Methods section of our revised paper (lines 155-161)

Was there any piloting conducted to assess contextual validity (I note that there was some to capture appropriate timings, so perhaps a little more information here would be good)? This task strikes me as one that will translate well across cultures (especially urban populations in India and especially since accuracy results are similar to the MTurk findings), and so I’m not overly concerned about these questions, but they are ones that came to mind as I read the manuscript. If the authors intend to use this task to more non-Western populations (especially non-urban ones), I recommend Holding et al. (2018), Hruschka et al. (2018) and Zuilkowski et al. (2016) as extremely useful sources discussing applying simple cognitive tasks across cultures.

We thank the reviewer for these very helpful suggestions. As mentioned above, we are currently using the BEAST across different populations. In our revised manuscript, we indicated a number of similarities in the data across these populations (lines 250-253). Furthermore, in our Methods section we now highlight that during piloting, we debriefed participants (lines 222-225). Finally, in our Discussion section, we highlight the minimal social context used by the BEAST, and we further nuance our results by adding these important references (lines 337-342).

Minor comments:

Line 38: The authors might want to consider including Wood et al. (2013) here.

Thank you for this suggestion. We added this reference.

Line 47: I’m not sure the references 27-29 are appropriate here, since I don’t think these studies gave children the choice of who they preferred to copy from. More appropriate references would be Price et al. (2017) and Wood et al. (2013) and references therein. However, the authors should also see Wood et al. (2016) for evidence directly contrary to their statement.

We have replaced these references with Price et al (2017) and Wood (2013). We also listed Wood (2016) as a counterexample.

Lines 86-88: I understand the sentient here, but as I’m certain the authors know India is an extremely diverse country (I have lived there!) and so I would rephrase this to be along the lines of ‘in some/most parts of India…’

We have now rephrased this to read ‘in many parts of India...’

The authors found that older adolescents were less likely to simply copy the estimates of others than younger adolescents. This is interesting, and somewhat counter to some overimitation work (e.g., McGuigan et al. 2011; Whiten et al. 2016), although it does fit with developmental work showing that older children are less likely to copy others than younger children (Carr et al, 2015). Do the authors have any ideas of why this might be?

Our data suggests that younger kids might be more likely to use ‘simple heuristics’ of staying and copying, rather than - potentially computationally more costly strategies of compromising / weighted averaging (cf. Moussaid et al 2013 PLoS One). We suspect that part of the discrepancies in the literature might be due to the study samples being from different populations, but also that social information use might strongly differ on the specific decision problem (experimental task) at hand. In our revision, we highlight this, and cite the suggested papers (lines 370-372).

Lines 303-308: Interesting – but most of these studies also used different tasks, which is likely to impact social information use (as the authors note in the introduction).

We fully agree that this makes the comparison less straightforward than it sounded. We added this qualification to make this point clear (lines 333-335).

Methods: The age range is 11-15. This strikes me as early/middle adolescence rather than adolescence in general.

Thank you for pointing this out. In our revision we added this qualification throughout (explicitly mentioning the age range in the abstract, and adding specifications in lines 44-45, 82-83, and 321-322).

Method/results: I wondered (and couldn’t find the answer anywhere) whether extended households were comprised of just children, parents and grandparents, or whether extended households included other members (aunts, uncles, cousins, etc.). If the former, then I wondered whether the authors had any thoughts on whether including ‘more extended’ households including other family members than grandparents would influence social information use? If the latter, did the authors examine whether number of extended household members had any effect on social information use?

Thank you for this suggestion. In addition to the presence of grandparent(s) in the household, we also have data on cohabiting aunts or uncles (that is, *present* or *not present*, not the exact number). Overall, our sample looks like this, with cells showing the number of participants:

|  | | Aunts / uncles | |
| --- | --- | --- | --- |
|  |  | **no** | **yes** |
| grandparents | **no** | 98 | 10 |
|  | **yes** | 106 | 42 |

Note that cohabiting aunts or uncles occurred much less frequently (for only 10 participants there were aunts/uncles but no grandparents). We also ran all analyses considering more detailed distinctions between each of these household types, but all lead to the same conclusion: social information is used more when provided by an adult, and household composition does not matter. We added the above table to the SI, and mention that our main results are robust to defining ‘extended households’ in various ways (lines 239-243).

Results: Although not significant, the results suggest that participants from nuclear households were actually more likely to information from an adult (compared to a peer) than those from extended households. Do the authors have any thoughts on that?

We agree that the data points into a potentially interesting direction opposite to our hypothesis. In our Discussion section (lines 392-399) we do highlight the absence of an overall effect (nuclear vs extended households) and possible reasons for this. However, we believe that the reader would best draw their own conclusions about this non-significant trend in our data. So, based on this comment we did not make any changes in our revised manuscript. We hope the reviewer is OK with this.

Results: Were there any diagnostics/assumption meeting conducted on the data?

Visual inspection of the paired differences for the paired t-test (line 265) looked normal; due to the large sample size, a KS-test detects marginally significant deviations from normality (*D*=0.080; *P*=0.071). That said, a (non-parametric) paired Mann-Whitney test confirms the results of the paired t-test (*P*=0.040). For the regressions fitted to S (Table 1, column 1) we draw the same conclusion (KS-test: *D*=0.060; *P*=0.044). For the logistic regressions (table 1 columns 2 and 3), the appropriate (logit) link function deals with the binary outcome variable. We now report these assumption checks in the Supporting Text, and refer to this in the Methods section of our paper.

Discussion: As noted above, I really applaud the assessment of non-traditional experimental psychology populations (i.e. not WEIRD). However, as should be done in in those studies, the authors should consider mentioning/discussing that these findings should be considered within the context of the population studied. For instance, the authors state that “Our results provide a deeper understanding into adolescents’ social information use and how.…” (lines 330-333). The authors might want to highlight that these findings need to be verified across other populations (unless this specific study is replicated across populations). They do this very briefly in the final paragraph (which is great, but I would like to see it a little more in the main discussion).

Thank you for bringing this up. We have rewritten this paragraph to reflect the fact that it is an empirical question whether our results would generalize to other (WEIRD and non-WEIRD) populations (lines 370-372). Furthermore, we double-checked the full text of our paper, and made this qualification where appropriate.

Lines 338-340: Additionally, adolescence brings the maturation of cognitive mechanisms including executive functions. It’s possible that the decrease in copying with age is in part a result of better equipped cognitive profiles, and thus a lower need to rely on others for information for this sort of task.

We fully agree with the reviewer. In our Discussion section (lines 367-372) we touch upon these points, adding the caveat that this might indeed depend on the task type.

Lines 341-350: I wonder whether the high frequency of extreme heuristics is a result of the quick nature of the task. That is, perhaps in a task with more time to develop strategies you might see more complex and more decision-making approaches.

Thank you for raising this point. Below we plot the relative frequencies of ‘copying’ and ‘staying’ across the 10 rounds of our task (Fig. RR1). The frequency of copying was highest in the first round, and slightly decreases over the periods of the task. Including the variable ‘round number’ in the regressions (table 1, columns 2 and 3) does not change any of the results (effects of ‘model type’ and ‘age’ remain significant). For the ‘stay’ heuristic there are no trends across periods. So it seems that we cannot conclude that extreme heuristics in general decrease. We prefer not to include these secondary explorations in the main text and in our revision we haven’t done so. If the reviewer insists we would be happy to include them though.

**Figure RR1.** Frequency of ‘stay’ (blue solid line) and ‘copy’ (red dashed line) over the course of the 10 rounds of the task. Data pooled across the conditions (the order of which was counterbalanced between participants).

Lines 363-365: Do the authors have any information of participant family values? If participants from nuclear families show similar such values to those in extended families (potentially indicating a recent shift), this argument would be presumably be supported.

Unfortunately we do not have these data. We now explicitly admit this fact (lines 395-399).

An interesting addition to this study would be to see if individual confidence predicted the adjustment to social information (and in relation to adult/peer nuclear extended). Other studies with relatively similar paradigms have shown that task confidence is a predictor of social information use (Morgan et al. 2012; Cross et al. 2017).

Thank you for this suggestion - we have added this potentially very interesting aspect to the discussion, including references to these two papers (lines 414-415).

References (in no particular order)

Thank you for providing the references!

Wood, L. A., Kendal, R. L. and Flynn, E. (2013) ‘Whom do children copy? Model-based biases in social learning’, Developmental Review, 33(4), pp. 341–356. doi: 10.1016/j.dr.2013.08.002.

Price, E., Wood, L. A. and Whiten, A. (2017) ‘Adaptive cultural transmission biases in children and nonhuman primates’, Infant Behavior and Development. Elsevier Inc., 48, pp. 45–53. doi: 10.1016/j.infbeh.2016.11.003.

Wood, L. A. et al. (2016) ‘“Model age-based” and “copy when uncertain” biases in children’s social learning of a novel task’, Journal of Experimental Child Psychology, 150, pp. 272–284. doi: 10.1016/j.jecp.2016.06.005.

Holding, P. et al. (2018) ‘Can we measure cognitive constructs consistently within and across cultures? Evidence from a test battery in Bangladesh, Ghana, and Tanzania’, Applied Neuropsychology: Child. Psychology Press, 7(1), pp. 1–13. doi: 10.1080/21622965.2016.1206823.

Zuilkowski, S. S. et al. (2016) ‘Dimensionality and the Development of Cognitive Assessments for Children in Sub-Saharan Africa’, Journal of Cross-Cultural Psychology. SAGE PublicationsSage CA: Los Angeles, CA, 47(3), pp. 341–354. doi: 10.1177/0022022115624155.

Hruschka, D. J. et al. (2018) ‘Learning from failures of protocol in cross-cultural research.’, Proceedings of the National Academy of Sciences of the United States of America. National Academy of Sciences, 115(45), pp. 11428–11434. doi: 10.1073/pnas.1721166115.

Morgan, T. J. H. et al. (2012) ‘The evolutionary basis of human social learning’, Proceedings of the Royal Society B: Biological Sciences, 279(1729), pp. 653–662. doi: 10.1098/rspb.2011.1172.

Cross, C. P. et al. (2017) ‘Sex differences in confidence influence patterns of conformity’, British Journal of Psychology, 108(4), pp. 655–667. doi: 10.1111/bjop.12232.

Whiten, A. et al. (2016) ‘Social learning in the real-world: “over-imitation” occurs in both children and adults unaware of participation in an experiment and independently of social interaction’, PLOS ONE. Edited by R. L. Kendal. Public Library of Science, 11(7), p. e0159920. doi: 10.1371/journal.pone.0159920.

McGuigan, N., Makinson, J. and Whiten, A. (2011) ‘From over-imitation to super-copying: Adults imitate causally irrelevant aspects of tool use with higher fidelity than young children’, British Journal of Psychology, 102(1), pp. 1–18. doi: 10.1348/000712610X493115.

Carr, K., Kendal, R. L. and Flynn, E. (2015) ‘Imitate or innovate? Children’s innovation is influenced by the efficacy of observed behaviour’, Cognition, 142, pp. 322–332. doi: 10.1016/j.cognition.2015.05.005.
